# Predicting Disease Related microRNA Based on Similarity and Topology

**DOI:** 10.3390/cells8111405

**Published:** 2019-11-07

**Authors:** Zhihua Chen, Xinke Wang, Peng Gao, Hongju Liu, Bosheng Song

**Affiliations:** 1 Institute of Computing Science and Technology, Guangzhou University, Guangzhou 510006, China; czhgd@gzhu.edu.cn; 2 School of Artificial Intelligence and Automation, Huazhong University of Science and Technology, Wuhan 430074, China; xk_wang@hust.edu.cn (X.W.); 18202718193@163.com (P.G.); 3 College of Information Technology and Computer Science, University of the Cordilleras, Baguio 2600, Philippines; liuhongju3@163.com; 4 School of Information Science and Engineering, Hunan University, Changsha 410082, China

**Keywords:** miRNA, network embedding, heterogeneous network, link prediction, topology information, machine learning

## Abstract

It is known that many diseases are caused by mutations or abnormalities in microRNA (miRNA). The usual method to predict miRNA disease relationships is to build a high-quality similarity network of diseases and miRNAs. All unobserved associations are ranked by their similarity scores, such that a higher score indicates a greater probability of a potential connection. However, this approach does not utilize information within the network. Therefore, in this study, we propose a machine learning method, called STIM, which uses network topology information to predict disease–miRNA associations. In contrast to the conventional approach, STIM constructs features according to information on similarity and topology in networks and then uses a machine learning model to predict potential associations. To verify the reliability and accuracy of our method, we compared STIM to other classical algorithms. The results of fivefold cross validation demonstrated that STIM outperforms many existing methods, particularly in terms of the area under the curve. In addition, the top 30 candidate miRNAs recommended by STIM in a case study of lung neoplasm have been confirmed in previous experiments, which proved the validity of the method.

## 1. Introduction

MicroRNAs (miRNAs) are a class of small endogenous non-coding RNAs with a length of approximately 20–24 nucleotides. miRNAs control the degradation and expression of messenger RNA through complementary base pairing [1]. Studies have shown that miRNAs play important roles in many biological activities, such as cell development, differentiation, apoptosis, metabolism, and signal transmission [2,3]. Furthermore, abnormalities in miRNA mutation and regulation are associated with many diseases. Therefore, identifying disease–miRNA associations can facilitate the study of disease pathogenesis and treatment options [4]. However, biological experiments are limited by high cost and long execution times. So developing a computational approach to identify potential disease–miRNA connections is imperative. This challenge is known as the link prediction problem in miRNA–disease networks [5,6,7,8,9,10,11,12,13].

Research predicting the links between diseases and miRNAs is primarily based on two aspects: (i) similarity networks, and (ii) machine learning models. Jiang et al. proposed the first calculation method for constructing a human disease–miRNA network and calculated the similarity score by accumulating hypergeometric distributions [14]. To improve their approach, the authors subsequently used Bayesian models to integrate multiple genomic data; they then calculated the functional similarity between genes on the basis of the biological knowledge that miRNAs regulate diseases through their target genes [15]. Bioinformatics researchers have proposed other network based computing models [16,17] for predicting the associations between miRNAs and diseases. Xuan et al. have calculated miRNA functional similarity on the basis of disease terms and phenotype similarity. They have used a weighted *k* nearest neighbors algorithm to prioritize miRNA–disease associations, in a method called human disease related miRNA prediction (HDMP) [18]. In [19], Chen et al. have proposed the random walk with restart for miRNA–disease association (RWRMDA) model, which identifies potential miRNA–disease pairs through random walks on miRNA functional similarity networks. Shi et al. have used miRNA–target associations, disease–gene associations, and protein–protein interaction networks to improve the RWRMDA model [20]. Xuan et al. have proposed a model for miRNAs associated with disease prediction (MIDP), which uses different node features based on random walks with restarts [21].

Machine learning methods also have numerous applications for predicting disease associated miRNAs [22]. In [23], Jiang et al. have proposed using a support vector machine (SVM) to mine potential miRNA–disease relationships. Their method involves using a feature set consisting of a miRNA functional similarity score associated with each disease, and a performance similarity score between a disease and other miRNA associated diseases. On the basis of miRNA expression information, Xu et al. have trained SVMs on miRNA target gene disorder networks to predict miRNA–disease associations [24]. Moreover, Chen et al. have proposed a semi supervised method based on regularized least squares, called regularized least squares for miRNA–disease association (RLSMDA) [25].

The methods described above integrate known miRNA–disease associations, disease–disease similarity datasets, and miRNA functional similarity networks to predict diseases without negative samples. However, methods based on similarity primarily rely on biological knowledge outside the network to calculate the similarity between nodes. To our knowledge, the extensive topological information between biological network nodes has not been utilized in similarity-based methods. Nevertheless, studies have shown that the numerous topological interactions between biological entities in heterogeneous networks are highly relevant to predicting associations [26,27].

In this study, we propose a prediction method called STIM, which uses information on similarity and topology. STIM can simultaneously predict the relationship between hundreds of diseases and hundreds of RNAs, thus serving as a systematic method to analyze correlations. The results of our evaluation via fivefold cross validation showed that STIM outperforms many previous methods. Moreover, 95% of the top 30 miRNA molecules predicted by STIM in a case study of lung neoplasm have been manually confirmed in our experimental studies.

## 2. Materials and Methods

**Figure 1:**
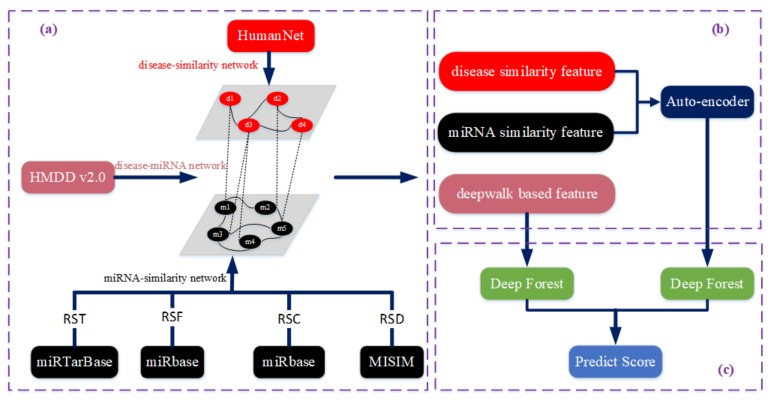
Workflow of STIM. (**a**) Network construction based on different resources. (**b**) Two kinds of feature extractions, one of which is passed through an auto-encoder. (**c**) Deep forest-based association prediction.

We propose a novel method, called STIM, to predict disease–miRNA associations. The framework for STIM is shown in Figure 1 and it contains three steps. First, a bilayer network with abundant information is constructed with different integrated data sources (Figure 1a). Second, given the bilayer network, two kinds of feature vectors are generated composed of similarity features and network embedding features guided by DeepWalk. In the experiment, the Deepwalk algorithm can be obtained from the deeplearning4j library (http://deeplearning4j.org/) [28]. Subsequently, for the similarity features, a corresponding feature vector is passed through an auto-encoder to obtain lower-dimensional features (Figure 1b). Third, given the two types of feature vectors obtained in last step, a prediction score is calculated based on the deep forest model, to predict the association between diseases and miRNA (Figure 1c).

### 2.1. Constructing the Bilayer Network

We first constructed a bilayer network—including a disease–miRNA association network, a disease similarity network, and a miRNA similarity network—which was integrated with various data sources. To construct the disease–miRNA association network, we downloaded known disease–miRNA associations from HMDD v2.0 [29], acquiring 6441 known disease–miRNA associations between 336 diseases and 577 miRNAs after filtering out duplicate records, as shown in Table 1.

To construct the disease similarity network, we downloaded data from the HumanNet database [30], which contains an associated log likelihood score (LLS) of each interaction between two genes or gene sets. As functionally similar genes tend to be associated with similar diseases, we calculated the disease similarity (DS) score to determine the edges among diseases. The DS score is calculated as follows:

(1) $$DS\left(d_{i},d_{j}\right)=\left\{\begin{array}{cc} \frac{\sum_{g_{1}\in S\left(d_{i}\right)}LLS\left(g_{1},S\left(d_{j}\right)\right)+\sum_{g_{2}\in S\left(d_{j}\right)}LLS\left(g_{2},S\left(d_{i}\right)\right)}{|S\left(d_{i}\right)|+|S\left(d_{j}\right)|}, & |S\left(d_{i}\right)|+|S\left(d_{j}\right)|\neq 0\\ 0, & otherwise \end{array}\right.$$

where $S(d_{i})$ and $S(d_{j})$ represent the gene sets related to diseases $d_{i}$ and $d_{j}$, respectively. $LLS(g_{1},S\left(d_{j}\right))$ is the log likelihood score between gene $g_{1}$ and gene set $S(d_{j})$. And $LLS(g_{2},S\left(d_{i}\right))$ is calculated in the same way. In a word, an edge exists if the DS score is greater than 0. Since there are 336 diseases in the disease–miRNA association network, we ultimately obtained a disease similarity network with 112,896 weighted edges based on the score calculated above.

To construct the miRNA similarity network, we collected four data sources altogether, including verified miRNA–target associations, miRNA family information, miRNA cluster information, and verified miRNA–disease associations. These were downloaded from miRTarBase, miRbase, miRbase, and MISIM, respectively. Specifically, for the miRNA–target associations, two miRNAs are connected if they share common targets. The edge weight (defined as RST) is the number of shared targets between miRNAs. For the miRNA family information (defined as RSF), if two miRNAs belong to the same family, their RSF value is 1, and otherwise 0. For the miRNA cluster information (defined as RSC), if two miRNAs belong to the same cluster, the RSC value of the two miRNAs is 1, and otherwise 0. For the miRNA–disease associations (defined as RSD), we get the RSD from MISIM. Finally, we combine the RST, RSF, RSC, and RSD to calculate the ultimate similarity score (RS) between miRNAs as follows:

(2) $$RS\left(r_{i},r_{j}\right)=\alpha \times RST\left(r_{i},r_{j}\right)+\beta \times RSF\left(r_{i},r_{j}\right)+\gamma \times RSC\left(r_{i},r_{j}\right)+\delta \times RSD\left(r_{i},r_{j}\right)$$

where α, β, γ, and δ are the corresponding parameters to adjust the weights of the four parts. Various values for these parameters have a different effect on the final prediction performance. In our evaluation, the best performance was achieved when α, β, γ, and δ were set to 0.2, 0.1, 0.2, and 0.5, respectively.

### 2.2. Feature Extraction and Selection

Feature engineering plays an important role in machine learning problems [31]. Feature engineering refers to the conversion of raw data into data that can be used for training. To obtain a better set of features, we constructed features based on the similarity information provided by external databases and the topology information inside the network [32,33]. This included two types of features, as shown in Figure 1b: (i) similarity features pertaining to disease and miRNA—i.e., the DS matrix and RS matrix calculated in last section; and (ii) DeepWalk-based features [34].

**Figure 2:**
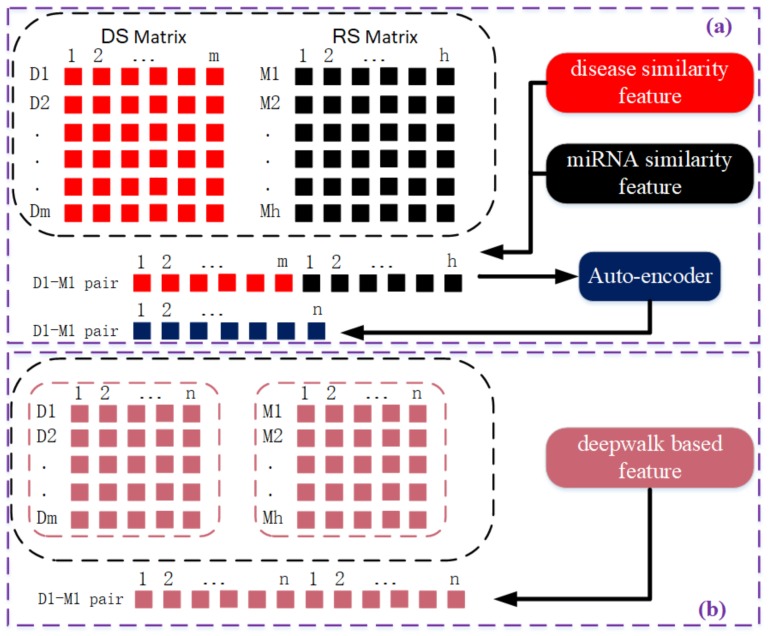
A schematic diagram of generating two kinds of eigenvectors. (**a**) the upper part: the similarity based feature vector: a n-dimensional disease-miRNA pair feature vector is obtained by the auto-encoder processing the dimension reduction of the cascaded m-dimensional disease feature vector and h-dimensional miRNA feature. (**b**) the lower part: the feature vector generated based on DeepWalk: a 2n-dimensional disease-miRNA pair feature vector is obtained by the cascaded n-dimensional disease feature vector and n-dimensional feature.

According to the DS matrix, the similarity feature for a specific disease is the corresponding row vector of the DS matrix, in which each element represents its similarity to other diseases. The length of each disease similarity feature vector is 336, corresponding to the number of disease nodes in the bilayer network. Similarly, according to the RS matrix, we can derive a miRNA similarity feature vector whose length is 577. In contrast to extracting similarity features from disease or miRNA similarity networks, DeepWalk-based features are extracted from the disease–miRNA association network using a network embedding algorithm called DeepWalk. Details for this algorithm can be found in the following section. In short, DeepWalk uses random walks on the association network to learn the latent representations of each node. As a result, each disease and miRNA node is represented as an n-dim feature vector. For each disease–miRNA pair, its feature vector is the concatenation of the disease vector and miRNA vector. In particular, the length of the feature vector derived from similarity-based features is the sum of the number of diseases and miRNAs, which is a very large sum. Therefore, an auto-encoder is used to reduce the dimensions of the features in the feature vector and automatically identify significant feature combinations for each disease–miRNA pair. Figure 2 shows the feature extraction process and how disease–miRNA pair feature vectors are generated. The parameters used in Figure 2 represent that m = 336 diseases; h = 577 miRNA; and n is the dimension of the vector. For the disease-disease DS,n is 336×336; while for the miRNA-miRNA RS, n is 577×577; and for the disease-miRNA pair, n is 336+577=913. Since the feature vector dimension of disease-mirna pair generated based on similarity is 913, which is relatively large, dimensionality reduction processing is required. After auto-encoder processed, the shape of disease-miRNA pair is reduced to 256. But For the deepwal-guided feature vector, the disease and miRNA nodes, n=128; the disease-mirna pair, *n* is 128+128=256 dimensions. The eigenvector dimension of the final disease-mirna pair is 2×n.

#### 2.2.1. Mining Network Topology Information Based on Deepwalk

DeepWalk, a deep learning method, learns latent topology information by randomly “walking” through the network, and outputs lower-dimensional vectors to represent the each node in the network. DeepWalk mainly consists of two parts. First, a random walk generator randomly and uniformly selects nodes and generates a fixed-length random walk sequence. For each node $v_{i}$, the random walk generator generates γ sequences of length *t*. Second, for each walk, the SkipGram algorithm is used to update the vector representation Φ of the nodes. The SkipGram maximizes the co-occurrence likelihood of the nodes that come into view within a window *w* to update the node’s representation vector Φ using independent assumptions as follows:

(3) $$P_{r}\left(\left\{v_{i-w},....,v_{i+w}\right\}\setminus v_{i}|\Phi \left(v_{i}\right)\right)=\Pi_{j=i-w}^{i+w}P_{r}\left(v_{j}|\Phi \left(v_{i}\right)\right)$$

where *w* in Equation (3) is defined as the size of the window, which is set as *w* = 5. Φ denotes the latent topological representation vector of the each node $v_{i}$ in the association network. Φ is represented by the matrix |V|*n, as shown in Figure 2, where |V| is the number of nodes and *n* is the dimensions of the representation vector for each node. To speed up training, $P_{r}$(v${}_{j}$|Φ(v${}_{j}$)) can be approximated using Hierarchial Softmax [35] by assigning each node to the leaves of a binary tree. Then, $P_{r}$(v${}_{j}$|Φ(v${}_{j}$)) can be calculated as follows:

(4) $$P_{r}\left(v_{j}|\Phi \left(v_{j}\right)\right)=\Pi_{l=1}^{log|V|}1/\left(1+e^{-\Phi \left(v_{i}\right)\Psi \left(b_{l}\right)}\right)$$

where $\Psi (b_{l})$ denotes the parent of tree node $b_{l}$, $b_{l}\in \left(b_{0},b_{1},b_{2},\ldots \ldots ,b_{log|v|}\right)$, a sequence of tree vertices used to identify the vertex $v_{j}$, where $b_{0}$ is the root node and $b_{log|v|}$ is node $v_{j}$.

After properly training, the output of DeepWalk is a latent topological representation vector for each node in the original network (i.e., an *n*-dimensional vector) as shown in the bottom of Figure 2.

#### 2.2.2. Constructing the Sample Space

As is shown in Figure 2, we concatenate the disease feature vector and miRNA feature vector to obtain the disease–miRNA pair feature vectors, which are fed subsequently into a deep forest model for link prediction. The number of disease nodes in the disease–miRNA association network is 336, and the number of miRNA nodes is 577. When constructing samples by splicing, the sample space capacity is 193,872. There are 6441 known disease–miRNA associations, regarded as positive samples in the training, whereas there are 187,431 unlabeled samples. The ratio between positive and unlabeled samples is thus 1:29. Statistically, there are only a small number of potential associations in unlabeled samples. By constructing samples, those that are potential associations can be diluted, so it is feasible to select a small number of negative samples from unlabeled samples to ensure that the ratio between positive and negative samples is 1:1. Here, we’re assuming that the number unknown positive associations is of the same order as the number of known associations.

### 2.3. Deep Forest-Based Association Prediction

We obtained the similarity matrix and topological vector matrix based on the disease–miRNA association network, as described in the previous section, and constructed the sample space by splicing. In order to combine the similarity information and the internal topology information of the network, we used the same models for predictions with the two sample sets along with weighted scores to form the final results [36,37].

**Figure 3:**
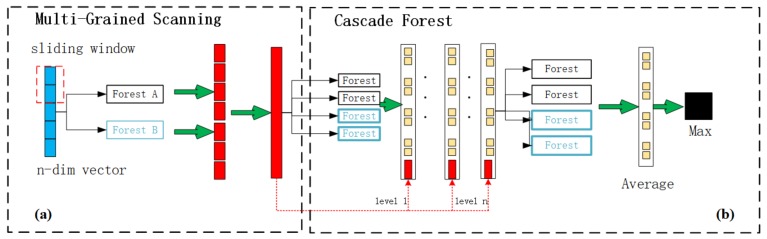
The schematic diagram of processing predictions. (**a**) a multi-granularity scanning is used to preprocess the input features. (**b**) the obtained eigenvectors are put into the cascaded forest for training.

In the experiment, by setting the dimension parameter *n* of the representation feature vector based on DeepWalk, we can control the dimensions of the samples, such that the generated sample features are beneficial for training. The dimensions of the sample based on the disease and miRNA similarity matrix are the sum of the number of network nodes, which is not only too large but too sparse and not conducive for training. To optimize the features and obtain advanced features, as described above, we used an auto-encoder [38] to learn the new lower-dimensional features. The self-encoder can be constructed with a deep neural network, which belongs to the coding. The encoder contains three layers, with 913, 512, and 256 neurons, and the decoder contains three layers, with 256, 512, and 913 neurons. For predicting associations, we selected the deep forest [39,40] model. This model has the same effect as a deep neural network, but with fewer parameters, simpler training, and good results on small samples. The deep forest model consists of two parts: multi-grained scanning, similar to convolutional neural networks, whereby multiple adjacent features are grouped; and a cascade forest, which integrates several weak classifiers into the forest and re-integrates them. Each layer is composed of four forests. Figure 3 shows the structure of the deep forest model.

We constructed two deep forest models with the same parameters to respectively train and predict two sample sets [41,42], as shown in the lower right corner of Figure 1. The main parameters include a window size of 100 and a classification category of 2. We relied on different sources of information to construct these two sample sets. To make full use of the two kinds of information, we adopted an integrated strategy to consider the prediction effects of the two different sample sets comprehensively [43,44]. The two predicted scores were obtained from different perspectives of the disease–miRNA association network. We assigned weights to the two scores into the final score as follows:

(5) s=λ∗s1+(1−λ)∗s2

where the parameter λ is used to adjust the proportion of the two part prediction result. It was set to 0.5 in the experiment.

## 3. Result

### 3.1. Evaluation Metrics

To illustrate the performance of the method at predicting potential associations between diseases and miRNA, we used five-fold cross-validation. For a specific disease *d*, *d* related miRNAs were randomly divided into five subsets. Four subsets were used as known information for training, and the remaining subset was used for testing. In what follows, we introduce three evaluation metrics for the performance of disease–miRNA association prediction.

The area under the receiver operating characteristic curve (AUC) is a global prediction performance indicator. We calculate the true positive rate (TPR) and the false positive rate (FPR) by varying the threshold, to obtain the receiver operating characteristic (ROC) curves:

$$\begin{array}{c} TPR=\frac{TP}{TP+FN}\\ FPR=\frac{FP}{TN+FP} \end{array}$$

where FN and FP are the number of negative and positive samples mistakenly identified, respectively, and TN and TP are the number of negative and positive samples correctly identified, respectively.

PRE is the accuracy of the positive predictions of the classifier for a specific disease. This concerns the quality of highly ranked results. It is calculated as follows:

$$PRE=\frac{TP}{TP+FP}$$

where TP and FP are the number of true positive and false positive samples with respect to a specific disease, respectively. Based on this definition, the larger the PRE value, the better the prediction accuracy.

REC is the ratio of positive instances that are correctly detected by the classifier for a specific disease, and concerns the quantity of the ranked results. It is calculated as follows:

$$REC=\frac{TP}{TP+FN}$$

where FN is the number of false negative samples with respect to a specific disease.

### 3.2. Performance of STIM

**Figure 4:**
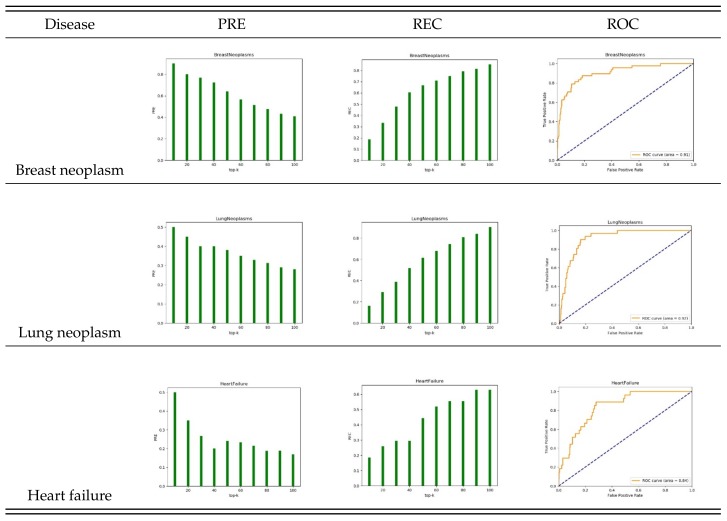
PRE, REC and ROC of different diseases by five-fold cross-validation.

To test the effectiveness of the algorithm, we selected diseases with known associations: breast neoplasm, lung neoplasm, and heart failure. We tested the performance of STIM predictions with five-fold cross-validation. We found that our method achieves good prediction performance for these diseases. Figure 4 shows the results in the top 10, 20, ..., 100 candidate miRNAs. The PRE and REC values of the breast neoplasm reached 90% at the top 10 and 82% at the top 100 with an AUC of 0.91. This means that 9 of the 10 predicted candidates were correctly identified. The highest PRE and REC values for lung neoplasm were 50% and 90%, respectively, with an AUC of 0.92. For heart failure the highest PRE and REC values were 50% and 63%, respectively, with an AUC of 0.84.

### 3.3. Comparison with Other Methods

We compared the proposed STIM with RWRMDA, HDMP, RLSMDA, and MIDP, which serve as advanced computational prediction models for discovering potential candidate miRNAs. Since many diseases collected from HMDD v2.0 have associations with only a few miRNAs, five-fold cross-validation of the performance may be insufficient. Therefore, we selected 15 characteristic diseases associated with more miRNAs.

As shown in Table 2, STIM achieved the best average AUC value with 0.874 and a maximum value of 0.913 (for lung neoplasms). This suggests that STIM works best for the lung neoplasms.

### 3.4. Case Study: Lung Neoplasm

To further demonstrate the reliability of STIM, we analyzed in detail its prediction accuracy for lung neoplasm, focusing on the top 30 miRNA candidates in Table 3. According to the databases dbDEMC 2.0 [45], miR2Disease [46], and related literature that is not used in the prediction process, the miRNAs with a higher score in the prediction results can be verified. dbDEMC is a differentially expressed miRNA database for human cancer. Using STIM, 23 of the top 30 candidate miRNAs could be found in dbDEMC. Similarly, miR2Disease is a manually compiled database designed to provide miRNA regulation abnormalities in human diseases. Using STIM, we found 6 of the 30 candidates in the miR2Disease database. In addition, we found that five of these miRNAs are associated with lung neoplasm, according to previous research [47,48]. These results demonstrate that STIM can effectively predict disease–miRNA associations.

## 4. Conclusions

We propose a disease–miRNA prediction method, called STIM, which uses machine learning models to combine similarity network information and network topology information. On the basis of a bilayer network, we integrated existing disease and miRNA similarity networks to obtain a similarity-based feature matrix. We then constructed a sample set via splicing. Further, we designed a self-encoder to map the original higher-dimensional features onto new lower-dimensional features to improve the prediction results. DeepWalk was used to mine topological information regarding the disease–miRNA association network. Then, we constructed a sample set from these DeepWalk-based features. Finally, the disease–miRNA pair feature vectors generated with two feature extraction methods were fed into two deep forest models to derive the final weighted predicted scores. Our evaluation showed that STIM achieved the best average AUC performance for 15 selected diseases, outperforming RWRMDA, HDMP, RLSMDA, and MIDP. Moreover, we confirmed the miRNA molecules recommended by MIST in a case study of lung neoplasm. Our evaluation thus confirmed the effectiveness, superiority, and reliability of the proposed algorithm, demonstrating that STIM is effective at predicting disease-related miRNA. We expect that this study will be of considerable help to future research on the pathogenesis of diseases.

## Figures and Tables

**Table 1:** Disease–miRNA associations network.

Node Type	Number
Disease	336
miRNA	577
Disease–miRNA associations	6441

**Table 2:** AUC comparison of different methods with specific diseases. (The best AUC value is shown in bold).

Disease	STIM	RWRMDA	HDMP	RLSMDA	MIDP
Acute myeloid leukemia	0.887	0.839	0.858	0.853	0.913
Breast neoplasm	**0.894**	0.785	0.801	0.832	0.838
Colorectal neoplasms	**0.855**	0.793	0.802	0.831	0.845
Glioblastoma	**0.857**	0.68	0.7	0.714	0.786
Heart failure	**0.829**	0.722	0.77	0.738	0.821
Hepatocellular carcinoma	**0.86**	0.749	0.759	0.794	0.807
Lung neoplasms	**0.913**	0.827	0.835	0.855	0.876
Melanoma	**0.857**	0.784	0.79	0.807	0.837
Ovarian neoplasms	0.89	0.882	**0.884**	0.909	**0.923**
Pancreatic neoplasms	0.909	0.871	0.895	0.887	**0.945**
Prostatic neoplasms	0.872	0.823	0.854	0.841	**0.882**
Renal cell carcinoma	**0.866**	0.815	0.833	0.839	0.862
Squamous cell carcinoma	**0.876**	0.819	0.82	0.849	0.87
Stomach neoplasms	**0.875**	0.779	0.787	0.797	0.821
Urinary bladder neoplasms	0.868	0.821	0.85	0.845	**0.897**
Average AUC	**0.874**	0.799	0.816	0.826	0.862

**Table 3:** Top 30 lung neoplasm-related candidates.

Rank	miRNA	Evidence	Rank	miRNA	Evidence
1	hsa-mir-130a	dbDEMC,miR2disease	16	hsa-mir-149	dbDEMC
2	hsa-mir-125b-2	Unconfirm	17	hsa-mir-15a	dbDEMC
3	hsa-mir-195	dbDEMC,miR2disease	18	hsa-mir-302a	Ref.[43]
4	hsa-mir-451a	dbDEMC,miR2disease	19	hsa-mir-99a	dbDEMC,miR2disease
5	hsa-mir-128-1	Unconfirm	20	hsa-mir-152	dbDEMC
6	hsa-mir-23b	dbDEMC	21	hsa-mir-708	Ref.[43]
7	hsa-mir-151a	Ref. [42]	22	hsa-mir-378a	Ref.[42]
8	hsa-mir-92a-2	dbDEMC	23	hsa-mir-339	dbDEMC
9	hsa-mir-302b	dbDEMC	24	hsa-mir-106b	dbDEMC
10	hsa-mir-193b	dbDEMC	25	hsa-mir-215	dbDEMC
11	hsa-mir-141	dbDEMC,miR2Disease	26	hsa-mir-130b	dbDEMC
12	hsa-mir-196b	dbDEMC	27	hsa-mir-302c	dbDEMC
13	hsa-mir-10a	dbDEMC	28	hsa-mir-296	dbDEMC
14	hsa-mir-429	dbDEMC,miR2disease	29	hsa-mir-320a	Ref.[43]
15	hsa-mir-328	dbDEMC	30	hsa-mir-20b	dbDEMC
